# Comparison between the for‐profit human milk industry and nonprofit human milk banking: Time for regulation?

**DOI:** 10.1111/mcn.13570

**Published:** 2023-10-13

**Authors:** Natalie Shenker, Jonathan Linden, Betty Wang, Claudia Mackenzie, Alex Pueyo Hildebrandt, Jacqui Spears, Danielle Davis, Sushma Nangia, Gillian Weaver

**Affiliations:** ^1^ Department of Surgery and Cancer, Imperial College London IRDB London UK; ^2^ The Human Milk Foundation Rothamsted Institute, Herts Harpenden UK; ^3^ Centre for Environmental Policy Imperial College London London UK; ^4^ Department of Neonatology Lady Hardinge Medical College New Delhi India

**Keywords:** breastfeeding, donor milk, exploitation, infant nutrition, markets, neonatology, paediatrics, regulation, WHO Code

## Abstract

Human milk (HM) is a highly evolutionary selected, complex biofluid, which provides tailored nutrition, immune system support and developmental cues that are unique to each maternal–infant dyad. In the absence of maternal milk, the World Health Organisation recommends vulnerable infants should be fed with screened donor HM (DHM) from a HM bank (HMB) ideally embedded in local or regional lactation support services. However, demand for HM products has arisen from an increasing awareness of the developmental and health impacts of the early introduction of formula and a lack of prioritisation into government‐funded and nonprofit milk banking and innovation. This survey of global nonprofit milk bank leaders aimed to outline the trends, commonalities and differences between nonprofit and for‐profit HM banking, examine strategies regarding the marketing and placement of products to hospital and public customers and outline the key social, ethical and human rights concerns. The survey captured information from 59 milk bank leaders in 30 countries from every populated continent. In total, five companies are currently trading HM products with several early‐stage private milk companies (PMCs). Products tended to be more expensive from PMC than HMB, milk providers were financially remunerated and lactation support for milk providers and recipients was not a core function of PMCs. Current regulatory frameworks for HM vary widely, with the majority of countries lacking any framework, and most others placing HM within food legislation, which does not include the support and care of milk donors and recipient prioritisation. Regulation as a Medical Product of Human Origin was only in place to prevent the sale of HM in four countries; export and import of HM was banned in two countries. This paper discusses the safety and ethical concerns raised by the commodification of HM and the opportunities policymakers have globally and country‐level to limit the potential for exploitation and the undermining of breastfeeding.

## Key messages

- The number and scope of human milk (HM) profit‐making companies is increasing globally
- Current regulatory frameworks for HM vary widely
- Products offered by private milk companies (PMCs) are generally more expensive than from HM banks
- Some PMCs are adopting marketing practices traditionally associated with breast milk substitute companies.
- The health benefits of HM‐based fortifiers compared to bovine milk‐based fortifiers are unclear and may be marginal

## Graphical abstract

Global regulations for the provision of human milk (HM) vary or are entirely absent. Private companies supplying HM‐derived products are now available in numerous countries, with multiple differences in operational models compared to nonprofit HM banks. This paper discusses the potential safety and ethical concerns raised by the commodification of HM and the opportunities policymakers have globally and country‐level to limit the potential for exploitation and the undermining of breastfeeding.

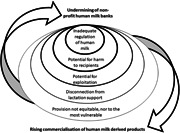

## 1 INTRODUCTION

As the creation of profit‐making ventures from another individual's body is prohibited under international law, regulations have developed over decades to limit trade in human‐derived products and the resulting harms. Frameworks now exist in national and regional law, produced by regulatory bodies, for the collection, production and distribution of a wide range of human‐derived products, including notably blood, tissues, organs and cells. Donor human milk (DHM) provided by a human milk bank (HMB) has been used for over a century to feed premature and otherwise vulnerable infants in the absence or incomplete supply of maternal milk, but is seldom been included in such regulatory frameworks (Klotz et al., 2022; Tyebally Fang et al., 2021; WHO, 2017). HMBs exist to screen, recruit and support milk donors, as well as process donated milk, usually through pasteurisation, alongside microbiological screening. Burgeoning evidence has led to the World Health Organisation recommending the use of screened DHM from a HMB as the first‐line feed after maternal milk for premature infants (Israel‐Ballard et al., 2019; PATH, 2017). However, DHM tends to only be used for the nutrition of the most extremely preterm infants (those born less than 32 weeks gestation or <1500 g birthweight); this means over 500,000 extremely premature infants globally currently have no access to DHM (Shenker et al., 2021). Where used in the context of optimal lactation support, maternal breastfeeding is supported by the availability of DHM (Kantorowska et al., 2016; Mondkar et al., 2022; Ponnapakkam et al., 2021; Wilson et al., 2018), and infant outcomes are improved in terms of reduced complications of prematurity, improved feed tolerance and avoidance of early supplemental formula (Mizuno et al., 2020; Ponnapakkam et al., 2021; Quigley et al., 2019).

Awareness of DHM provision and the ability to donate milk can help to support positive perceptions of breastfeeding in the wider national consciousness. However, a lack of investment in HMB services and innovation has created an environment where medical and public demand for DHM often exceeds supply (Battersby et al., 2018). As a result of this, in addition to awareness of the potential health impacts of exclusive human milk (HM) diet in neonates, profit‐making companies (PMCs) have been able to establish operations over the last 15 years in countries that lack regulatory frameworks that protect both providers and recipients, operating independently of lactation support services that underpin nonprofit HM banking (Reimers & Coutsoudis, 2021). PMCs have tended to remunerate women for their milk and create HM‐derived products that are then sold to neonatal units, but little research has been published on their work.

The COVID‐19 pandemic highlighted vulnerabilities in service provision and emergency preparedness within the sector (Shenker et al., 2021). Pandemic‐related challenges to service provision include insufficient donors, pre‐screening disruption, DHM availability and lack of logistics, communication, safe handling and contingency planning. These pressures led to the creation of the Global Alliance of Milk Banks and Associations (GAMBA) in an attempt to identify and address evidence gaps and provide support to milk banks facing these pressures (Shenker et al., 2020). By facilitating greater communication and information sharing, the global impact of for‐profit HM commercialisation became apparent and was identified through a series of webinars as a key pressure on nonprofit HMBs.

Although assessments of individual companies' practices have been published (Newman & Nahman, 2020), to date, no formal analysis of the difference between for‐profit companies (FPCs) and nonprofit HMBs has been conducted. Neither has there been a global scoping project of funding models and regulatory frameworks with regard to HM products. This study aimed to provide an overview of the current for‐profit and nonprofit HM sectors through an analysis of business models to identify risk and safeguarding considerations. Furthermore, an assessment of the key ethical concerns raised throughout the global HM supply chain was conducted in addition to a survey of country‐level regulatory frameworks that aim to protect mothers and infants.

## 2 METHODS

### 2.1 Survey of the global alliance of milk banks and associations

On March 17, 2020, a Virtual Collaboration Network of global milk bank leaders was formed, aiming to share learnings and actively discuss COVID‐19‐specific challenges and mitigation strategies (Shenker et al., 2021). In early 2021, members democratically decided to move towards creating a formal organisation. The GAMBA is now composed of over 150 members from 45 countries, including milk bank leaders, nongovernmental organisations and academics. Analysis of HM commercialisation was set as one of the early research priorities.

A survey was therefore conducted of GAMBA members, which at the time meant 95 milk bank leaders from 35 countries were contacted. The 44‐question survey was designed on Qualtrics and sent to the members of GAMBA for completion within the month of February 2021. The aim of the survey was to gather more information about the activity of FPCs globally and the DHM market generally; questions were directly about within‐country PMC activity, covering products, milk providers, provider remuneration, marketing and distribution channels. The survey also gathered data on the wider context of the HM market, nonprofit scope of operations and country‐specific regulations. The survey included a combination of tick box responses and open‐ended questions. A copy of the survey questions is included in Supporting Information: File 1.

### 2.2 Business model canvas (BMC) framework

Analysis of the business models within the HM industry was conducted using the BMC framework as a starting point. The BMC is a strategic management template used to examine business models (Osterwalder et al., 2005). The framework highlights important points of comparison such as value proposition, product, price and funding, customer/donor relationships, regulations and marketing. Alongside this, the evolution of the HM industry and each company's position within it was analysed, as this is not included within the scope of the BMC.

Prices of HM‐derived products were analysed where data was found; however, cost and revenue structures for the companies were not available. These are important resiliency measures for companies and warrant future investigation. The six PMCs, five trading and one developing premarket products, were identified from the GAMBA survey. It is noted that four of the companies are based in the United States, and, as such, are unlikely to be representative of all business models globally. However, the sample firms do provide a range within the sector in terms of size and activity. To draw meaningful insight into the whole market, a comparison was completed in the same manner for six nonprofit organisations. The milk banks selected represent different regions and their operating models across a spread of criteria, including resource setting and longevity; for example, Mother's Milk Bank in San Jose has been in operation since 1974, whereas the Da Nang HMB in Vietnam opened in 2017 and serves as a model for many HMBs that have opened across the country.

Research into these organisations was conducted primarily online using the company websites and web searches, which led to news articles, research papers and social media [example search terms, ‘for‐profit breast milk companies’, ‘for‐profit milk banks globally’, ‘human milk banks’, ‘prioritising patients in neonatal units’, ‘cost of humavant’, ‘price of humavant’, and ‘human milk export’].

### 2.3 Business model analysis

General online search engines were accessed with key search terms (e.g., ‘Human Milk Regulations’, ‘Human Milk Markets’, ‘Human Milk Donations and Exploitation’, ‘Human Milk Access’, ‘Best Practice Statements’, ‘Ethical Best Practice Statements’, ‘Ethical considerations for human milk banks’, ‘Equitable access for human milk’, ‘WHO guidelines for human milk banks’, ‘NICE Guidelines for human milk banks’, ‘Modern Slavery’, ‘Modern Slavery Human Milk Banks’, ‘Modern Slavery in Supply Chains’, ‘Preventing Modern Slavery’, ‘Exploitation in Supply Chains’, and ‘Human Milk Exploitation’) to research concerns regarding modern slavery and exploitation in supply chains relevant to HM. From this, potential areas of vulnerability were identified for HM donors, providers/donors and recipients. The same key terms were further utilised in scientific search engines to locate journal articles addressing ethical concerns and specific examples of exploitation of FPCs. Data from the literature search were cross‐referenced against themes identified in the open‐ended questions to check for potential contradictions and verification by the independent research in this study and to understand the scope of potential consequences for nonprofit HMBs and public health. From the identification of these areas of concern, a list of recommendations was compiled to act as a guide for the formulation of best practice statements.

### 2.4 Data analysis

Survey responses were collated using Microsoft Excel (Microsoft) and screened for partial responses. Duplicate entries were removed, and survey data from multiple HMBs within a single country were checked by two researchers who confirmed duplications of identical data and resolved any discrepancies in data through direct contact with the milk bank leader who had completed the survey. A descriptive analysis was then conducted by identifying themes related to the impact on nonprofit HMBs by PMCs or of potential public health concerns, in addition to internal discussions by the research team. Data related to regulatory frameworks were mapped geographically using publicly available software (SlideLizard®, https://slidelizard.com).

### 2.5 Ethics statement

Institutional ethics board waived ethics approval for this project as it came under a service evaluation, and no identifiable data was recorded. Participants gave informed consent before completing the survey.

## 3 RESULTS

### 3.1 Survey overview

Forty‐eight responses were received within the month of February 2021 and represented nonprofit HMB leaders from 29 countries (Supporting Information: Table 1), providing insight into a range of milk banking practices globally. Of respondents, 19 worked for a HMB funded directly by the Ministry of Health in their country, 17 for a milk bank funded regionally or in a local health care service, seven worked within an independent milk bank, four were neonatologists with clinical oversight for one or several milk banks and one represented an NGO with a specific focus on supporting milk bank infrastructure.

### 3.2 Value proposition and products

PMCs are distinguishable through two main features: innovative technology and provision of distinctive products in addition to pasteurised HM. The value proposition that they advertise is an investment into research and development (R&D) and technology which enables unique products to be produced to benefit infants. Four of the six FPCs produce nutritional fortifiers, which are made by processing HM to create specific calorific and nutrient formulations per ounce of product. Some companies, including Prolacta, Ni‐Q and Medolac, provide a range of fortifiers that differ by nutrient content. As preterm infants may not be able to consume large volumes of milk and cannot regulate energy intake, these products provide concentrated nutritional intake when mixed with mother's own milk or DHM. In addition, all six of the FPCs produce ready‐to‐feed pasteurised HM. PMC advertise the specific nutrient ratios of their products, which is enabled by technology, often patented; nonprofit HMBs generally do not routinely determine the nutritional content of DHM.

Another notable trend in HM products is related to storage. Most HMBs pasteurise DHM to a nonconcentrated form that is frozen and thawed before use. In the for‐profit market, Prolacta and LactaLogics are competing with the same ready‐to‐feed products that require freezing, and Prolacta's fortified products also require freezing. Newer FPCs, such as NeoLacta and sister company NeoKare, are developing shelf‐stable products (either in dried or liquid form) which are promoted as reducing the costs of creating the product and therefore the price to customers. These stable products will also be cheaper for hospitals to store and may be easier for practitioners to administer as they do not require thawing, although concerns have been raised regarding a loss of immune activity (Lima et al., 2017). Nonprofit milk banks, as demonstrated by Marmande Milk Bank, the largest HMB in France, also have innovated in this area by producing freeze‐dried milk with a shelf life of 18 months. There may be utility in terms of easier distribution via postage, although frozen products are easy to administer in NICUs, but the greatest potential need for shelf‐stable products would be for orphaned and other infants without access to maternal milk in disaster relief areas where freezers are unavailable and could be adequately supported through the nonprofit sector.

Tables 1 and 2 provide a comparison of operational models in the for‐profit and nonprofit sectors, including company details, regulators, customers, products produced and provider/donor recruitment processes.

**Table 1:** Overview of the practices of profit‐making human milk companies.

Attributes\Company	Prolacta	NeoLacta	NI‐Q	LactaLogics	Neokare	Medolac
*Company overview*						
Location	California, USA	Bangalore, India	Oregon, USA	Florida, USA	Redditch, UK	Nevada, USA
Date founded	1999	2016	2014	2012	2020	2009
Legal structure	Private	Private	Private	Co‐operative	Private	Public Benefit Corporation
Funding source	$78 mm (VC)	Unknown	$2.8 mm (source unknown)	Unknown	Unknown	Unknown
Revenue	$45.40 million (2019)	Unknown	Unknown	Unknown	Unknown	£4 Million
Markets	USA, Europe, Asia	India	USA	USA	UK	USA
Regulator	FDA	Unregulated	FDA	FDA	FSA	FDA
Customers	NICUs and the public	NICUs	NICUs and the public	NICUs and the public^a^	NICUs and the public	NICUs
*Products*						
Frozen human milk	✓	✓	✗	✗	✓	✗
Frozen human milk fortifier	✓	✗	✗	✗	✗	✗
Shelf‐stable human milk	✗	✗	✓	✓	✗	✓
Shelf‐stable human milk fortifier	✗	✗	✗	✓	✗	✗
Dried human milk fortifier	✗	✓	✗	✗	✓	✗
Dried human milk	✗	✓	✗	✗	✓	✗
Price (USD/OZ)	>$175/oz for fortifier	$12.38−45.38/oz	$6.50/oz	Unknown	$7.35/Oz	Unknown
*Donors*						
Equipment provided? For example, breast pumps?	✗	✓	✗	✗	✗	✗
Financial remuneration?	✓	✗	✓	✓	✓	✗
Is donor blood screened?	✓	✓	✓	✓	✓	✓

^a^ Not yet commercially available.

**Table 2:** A comparison of nonprofit human milk banks.

Attributes\Company	Mother's milk bank	Lifeblood red cross milk bank	Marmande milk bank	The human milk bank (Northern Ireland)	Da Nang HMB	HMB Sion Hospital
*Milk bank overview*						
Location	San Jose, USA	Sydney and Adelaide, Australia	Bordeaux and Marmande, France	Enniskillen, N. Ireland	Da Nang, Vietnam	Mumbai, India
Date founded	1974	C.1930	1955	2000	2017	1989
Legal structure	Nonprofit organisation	Charity	State Run	State Run	State Run	State Run
Funding source	Philanthropic donations	Government	Government	Government	Government	Government
Revenue	Not available	Not available	Not available	Not available	Not available	Not available
Markets	USA	Australia	France	Ireland	Vietnam	India
Regulator	FDA Licensed tissue bank	FSANZ	ASNM	FSA	Not available	FSSAI
Customers	NICUs and the public with doctor's prescription	NICUs	NICUs	NICUs and the public with doctor's prescription	NICUs and the public with doctor's prescription	NICUs
*Products*						
Frozen human milk	✓	✓	✓	✓	✓	✓
Frozen human milk fortifier	✗					
Shelf‐stable human milk	✗					
Shelf‐stable human milk fortifier	✗					
Dried human milk fortifier	✗					
Dried human milk	✗					
Price (USD/OZ)	$3.75/oz	Not available	~$3/oz	Not available	Not available	Not available
*Donors*						
Equipment provided? For example, breast pumps?	✗	✗	✓	✓	✗	✗
Financial remuneration?	✗	✗	✗	✗	✗	✗
Is donor blood screened?	✓	✓	✓	✓	✓	✓

Abbreviations: HMB, human milk bank.

### 3.3 Price and funding

The comparison of price is difficult as this information is not readily disclosed in the HM market and product offerings differ. Products offered by PMCs are generally more expensive than HMBs as fortifiers are a concentrated product. This processing requires more input HM, provider payments, equipment, research, screening and testing, which results in higher costs of production. In the six PMCs analysed, prices ranged from $6.60 to $175/oz (Anderson, 2020), depending on the calorific formulations. Acknowledging that these are different products, nonprofit and government‐run HMBs provide DHM for approximately $3−$5/oz in the United States, $3/oz in Italy and France and $4−$7/oz in the United Kingdom. Furthermore, we note that there is a downward trend in prices in the for‐profit market as new competitors are producing more affordable and accessible products. An example is Medolac, which publicly noted the high cost of traditional frozen DHM, but since the analysis was completed has ceased trading. The existence of these cost analyses suggests that prices are an important consideration for hospitals in terms of assessing cost‐effectiveness and potentially limits reaching a wider market, including the public.

An additional area of contrast between the for‐profit and nonprofit sectors is funding. FPCs are supported by private capital, which allows them to raise large amounts of funding—Prolacta has received $78 m (Crunchbase, 2022) from venture capital (VC) and Ni‐Q has received $2.8 m (Crunchbase, 2017). The VC firms invested in Prolacta, such as Health Evolution Partners and Alta Partners, invest in other health care, health technology and biotechnology companies, so would be able to provide advice, collaboration, networking opportunities and publicity for Prolacta. This funding further facilitates their technology and R&D value propositions, along with significant litigations between competitors (Anderson, 2020; Bloomberg, 2020; CPI, 2020).

When asked in the survey to describe sources of HMB funding, results showed that funding for the nonprofit sector primarily comes from central government (direct from Ministries of Health) or local health care provision (individual hospitals or regions). Of the 43 responses providing data from 26 countries, direct government funding or reimbursement from directly supported hospitals funded services in 25 countries. Health care funding was supplemented with philanthropic funding into milk bank services, or standalone charities that provided additional milk bank provision, in 13 countries. The development of one country's milk bank services was reported to be solely philanthropically funded. Health insurance supported the cost of access to DHM in three countries, but only if parents had that particular package. In five countries (the United States, South Africa, Philippines, Thailand and Vietnam), costs may be passed on to parents by the hospital. No HMB received direct funding from parents or private capital.

### 3.4 HM provider/donor recruitment and remuneration

PMCs recruit milk providers through a range of routes that are country and culturally dependent. These principally include digital channels, counsellors in private hospitals and direct contact with village elders in rural communities (Newman & Nahman, 2020). In the United States, one PMC has also partnered with NICUs that do not have their own milk bank or close links with an HMB to recruit HM providers, managing health screening, serology tests and milk collections (Prolacta). Hospitals recruit mothers in the community who have extra breast milk and they receive $1/oz for donor referrals (Yuma_Regional_Medical_Center, 2018); it is not clear whether these hospitals receive HM products in return. Analysis of the costs and benefits of partnering with PMCs will be required to ascertain this, which will differ by context.

Further differences exist between PMCs and nonprofit HMBs in the way they interact with donors through the recruitment process. PMCs tend to use rapid questionnaires with yes/no answers that allow them to screen and categorise providers quickly. HMBs generally use more open communication channels, prioritising conversations instead of or in addition to closed answer questionnaires. Questionnaires are extremely cost‐efficient as they allow the rapid recruitment and categorisation of donors according to a limited number of criteria at almost no cost. Nonetheless, mothers often need lactation assistance, mental health support and specific follow‐up help according to individual needs and concerns, which cannot be identified through questionnaires. PMCs do not provide in‐house breastfeeding support to mothers who face difficulties lactating, nor are they integrated into community‐based support services, unlike HMBs which do offer ongoing breastfeeding support.

For‐profit milk banks often compensate women financially to incentivize donations and compensate for time and travel expenses (Table 1). Prolacta, Ni‐Q, NeoKare and LactaLogics make payments according to the volume of HM donated. Although PMCs state that financially remunerating donors can increase the HM supply for infants and fairly compensate women for their effort, the introduction of financial motivation introduces ethical and practical concerns raised by the global HMB community and collated in Table 3. HMBs surveyed do not offer any financial compensation, but individual milk banks may pay back travel expenses incurred by donors travelling to get blood tests or costs of breast milk storage bags.

**Table 3:** The principal potential harms of human milk commercialisation.

Theme	Potential harm
Equity	Access based on ability to pay, which can lead to families being charged over $3000 per month. High costs to hospitals for human milk‐derived products, which may be over 100‐fold that of alternatives. Potential to limit donors to nonprofit/government HMBs, with potential for reduced DHM availability. Lack of tracking and tracing and potential pooling from hundreds of providers limits ability of Muslim parents to consent.
Safety	Little or no safety or efficacy data for new products, and available data may not be in comparable populations. Comprehensive provider screening may not happen, for example, omitting details of increased prion risk or travel history. Payment by minimum volumes risks milk being adulterated with water or other species milk. Payment after microbiology tests may result in flash pasteurisation or scalding by the provider before collection. Lack of oversight by experts in microbiological or serological screening. Paying for product could introduce safety breeches, for example, it is reheated by parents or not used within the time limits
Exploitation	Mothers may sell their milk rather than feed their own infants No or minimal lactation support to help establish a full milk supply or recover from other infant feeding issues Mothers may hyperlactate, with short‐ and long‐term risks to their health. Milk banks should be aiming to help mothers manage an uncomfortable or unwanted oversupply down, but payment by volume can introduce conflict. Minimum volumes expected by for‐profit companies tend to be high. Providers may be penalised financially if they do not meet the minimum volume, by being asked to pay for courier services as well as not being paid. For‐profit model means companies have few ethical safeguards to limit exploitation, particularly in low resource settings.
Disconnection from support	Milk banks exist within hospital or community lactation support, ensuring maternal breastfeeding is prioritised and DHM is not used inappropriately to undermine maternal lactation For‐profit companies tend to not provide lactation support for milk providers to manage common issues such as mastitis, support with medication use and donation and so forth.
Impacts on breastfeeding perceptions	Undermines importance of maternal milk and direct breastfeeding Confuses WHO‐led public health messaging that DHM should only be used in the absence of maternal milk Undermines nonprofit/government‐led sector providing community milk under health care professional oversight

*Note*: These are general principles, some already applicable to the companies already operating, some that may affect future providers.

### 3.5 Screening

All PMCs and HMBs studied include blood screening of donors before milk is accepted as a critical control point. Screening at this stage prevents ineligible milk from entering the processing site. It also identifies ineligible donors before they take on the task of expressing milk for donation and avoids associated costs of storing and transporting milk that would ultimately not be suitable for processing.

However, to ensure that the resulting products are safe to use, further testing of the milk is required on receipt at the processing site. The highest standards of testing, as detailed in guidelines such as the NICE Clinical Guideline #93 (NICE, 2010), involve screening and then processing each batch of milk from each donor. HMBs rely on accurate labelling and safe storage of containers as well as the trust relationship established with each donor during screening. More sophisticated forms of tracing of this process are conducted by Prolacta, in which DNA matches each batch back to the donor to ensure origin (Hartmann, 2017), potentially because of the additional risk of contamination with other milk when financial remuneration according to the volume of milk is introduced. Other forms of screening by PMCs are considered proprietary and cannot be discussed herein.

New technology has also developed as a solution to manage milk banking processes and ensure high levels of safety. Several HMBs in the United Kingdom utilise Li‐LAC, a software‐as‐a‐service platform developed by Savant Ltd., which delivers a ‘centralised track‐and‐trace control system’ to monitor the flow of milk through the system. As well as improving in‐house efficiency and tracking, such systems could be used to support stocking and logistical challenges for relatively minimal cost. However, such sophisticated systems are not deployed widely.

### 3.6 Processing

A clear distinction can be drawn between the products produced by PMCs and those produced by HMBs. Five of the six analysed HMBs exclusively produced pasteurised DHM without fortification, in which the processes of pasteurising, rapidly cooling and then freezing the resultant milk is a global standard (Moro et al., 2019). This technique is not limited to nonprofits and is used by PMCs (Prolacta, 2022); however, it would appear that this sequence of pasteurisation and freezing is the primary, even sole, process for many nonprofit HMBs.

The PMCs studied are broadly processing HM to deliver shelf‐stable products, either liquid or powdered (Table 1), rather than frozen products. However, drawing a detailed comparison of the methods used between these firms proves challenging as exact details are not publicly available, likely as they form part of the IP and USP of each brand. Where pasteurisation is deployed by PMCs, often before further processing steps, a range of different temperatures and time periods are described, with claimed benefits including the retention of important proteins and immune factors, as well as increased safety (Ni‐Q, 2022).

Several HMBs, including the Marmande Lactarium in France and others in Eastern Europe, produce a dried shelf‐stable powder from DHM. The motivation behind this innovation is to facilitate easier transportation, as the HMBs are located a distance from the hospitals they serve (Arnold, 1994). Logistical issues were a major theme of the challenges faced worldwide by HMBs, and investment in technology could help to safeguard supplies (Shenker et al., 2021).

### 3.7 End‐users

The primary market for PMCs comprises neonatal intensive care units (NICUs), focusing on providing products for use in infants who are born premature or low birthweight. Some PMCs are also selling products directly to families, outside of the hospital context (Ni‐Q, NeoLacta and Neokare). Although four out of six PMCs market their product for ‘infants who need it’, HM products are being sold outside of the hospital context and to full‐term babies at home (Neokare, 2021; Newman & Nahman, 2020). The survey responses showed this was happening either directly by the PMC to parents or via intermediaries in five countries: the Philippines, India, the United States, the United Kingdom and Germany.

In comparison, HMBs are usually established within a hospital or health care services and so provide DHM for that hospital and/or others in the country. Some HMBs also provide DHM directly to patients if it has been prescribed (e.g., Mothers' Milk Bank San Jose, Western Trust HMB and Da Nang HMB), and others provide surplus DHM to families under health care referral free at the point of need (e.g., Hearts Milk Bank). The consideration of the end user of DHM is important as it raises concerns of whether infants who are most vulnerable have equity of access, and whether families able to pay for DHM will gain enhanced access.

### 3.8 Marketing

Both PMCs and nonprofit HMBs are using their social media channels to engage with and recruit prospective milk providers/donors, with no significant differences identified in the way both types of organisations interact with parents. Organisations such as NeoLacta (India) use WhatsApp as their main communication channel with families and recipients' parents. A common practice is including a direct link to a WhatsApp chat on their corporate website. Communication channels also vary across geographical regions, with US, European, Australian and South American HMBs having strong social media presence, particularly within the last 5 years. Nonprofit HMBs in Asia, such as Da Nang (Vietnam) and HMB Sion Hospital (India), have a limited online presence, with no social media accounts or other forms of online marketing channels found, suggesting they use offline marketing channels or direct relationships with health care systems. Survey responses supported the finding that greater awareness might stretch small HMB teams, leading to more donors than could be recruited.

For HMBs in Europe and the United States, Facebook appears to be the most used social media platform to interact with the public, with 83% of PMCs and 57% of the nonprofit HMBs having a Facebook account. Facebook is followed by LinkedIn, Twitter, Instagram and YouTube. Prolacta, for example, uses its social media channels to share articles and statistics on the importance of HM, as well as to promote internal webinars on topics like ‘How to successfully onboard an Exclusive HM Diet in your NICU’ or events such as the ‘Family Health Festival’ or the ‘Prolacta Breakfast Symposium’. Prolacta also has a YouTube channel, with some of its videos accumulating over 220,000 views. The company uses YouTube to both share personal stories of babies in the NICU and provide specific information on Prolacta's supply chain and tips on how to freeze and store breast milk.

Survey reports highlighted PMCs adopting practices traditionally associated with breast milk substitute companies. These strategies include heavily marketing their products into both private and state neonatal units (India, the United Kingdom, the United States and Germany), providing funding for conferences and travel/hospitality for health care professionals (the United States and the United Kingdom), offering products to health care professionals with initial discounts (the United Kingdom and the United States), funding parent‐led advocacy groups (Europe, the United States and the United Kingdom), sponsoring training programmes for junior neonatologists (India), offering presents to parents (Philippines and Myanmar) and offering a significant financial prize for trainee essay competitions (India). PMCs were reported to have offered funding to health care professionals for clinical trials in seven countries (the United States, India, Israel, Germany, the Netherlands, Sweden and the United Kingdom), but were only taken forward in the United States, the United Kingdom and India. Government or charitably funded HMBs are unable to compete financially, and the impact on health care professionals of both direct and indirect marketing despite the absence of efficacy data in vulnerable patient populations is an area of outstanding research (Chartres et al., 2016; DeJong & Steinbrook, 2018; Fabbri et al., 2018; Khabsa et al., 2020; Lundh et al., 2017).

### 3.9 Regulation

**Figure 1:**
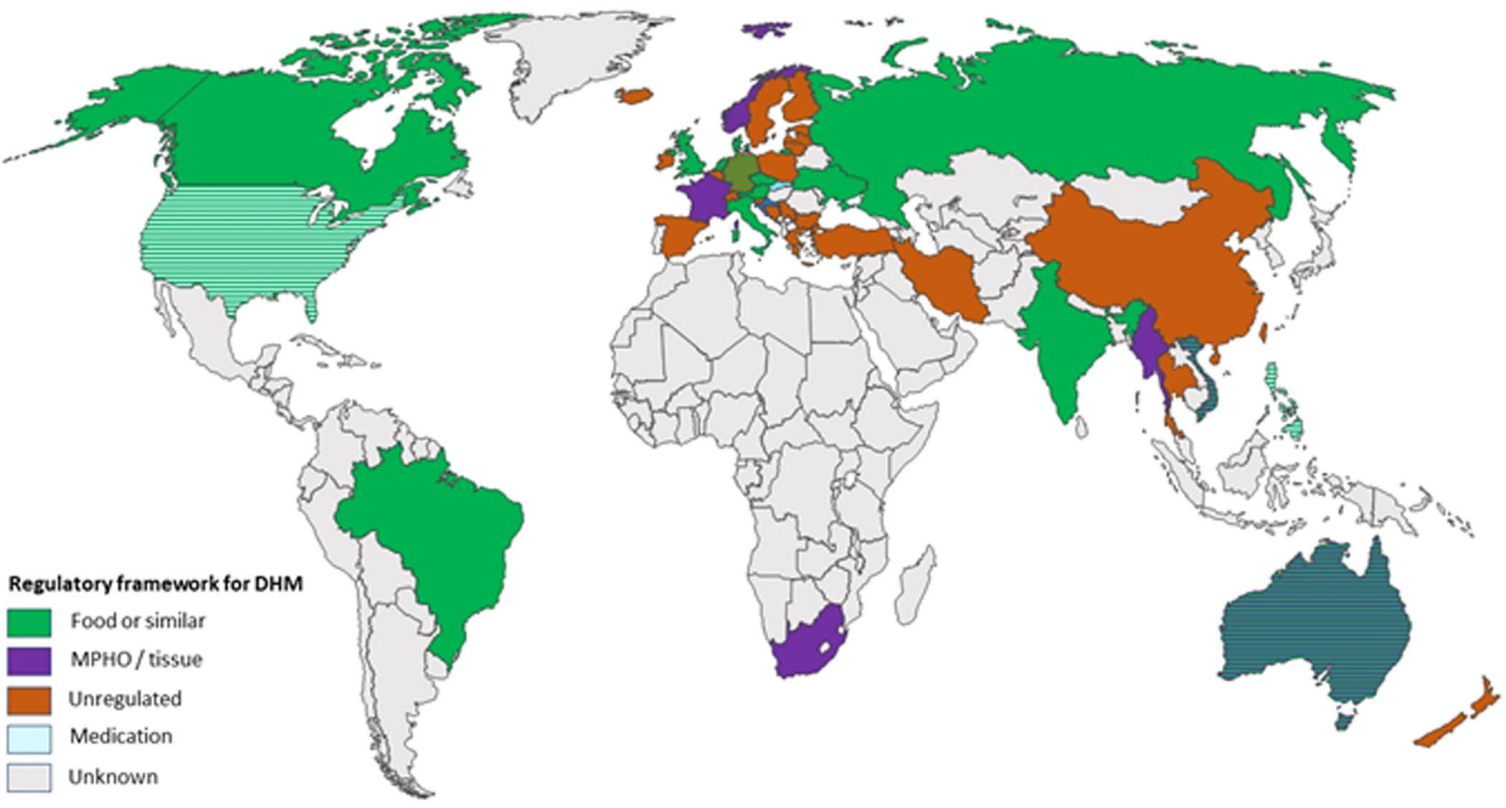
Regulatory frameworks for donor human milk/human milk products globally. Striped indicates where two frameworks co‐exist for different products (e.g., the United States and Australia), or where some regions of a country have regulations and others do not (e.g., Germany).

**Figure 2:**
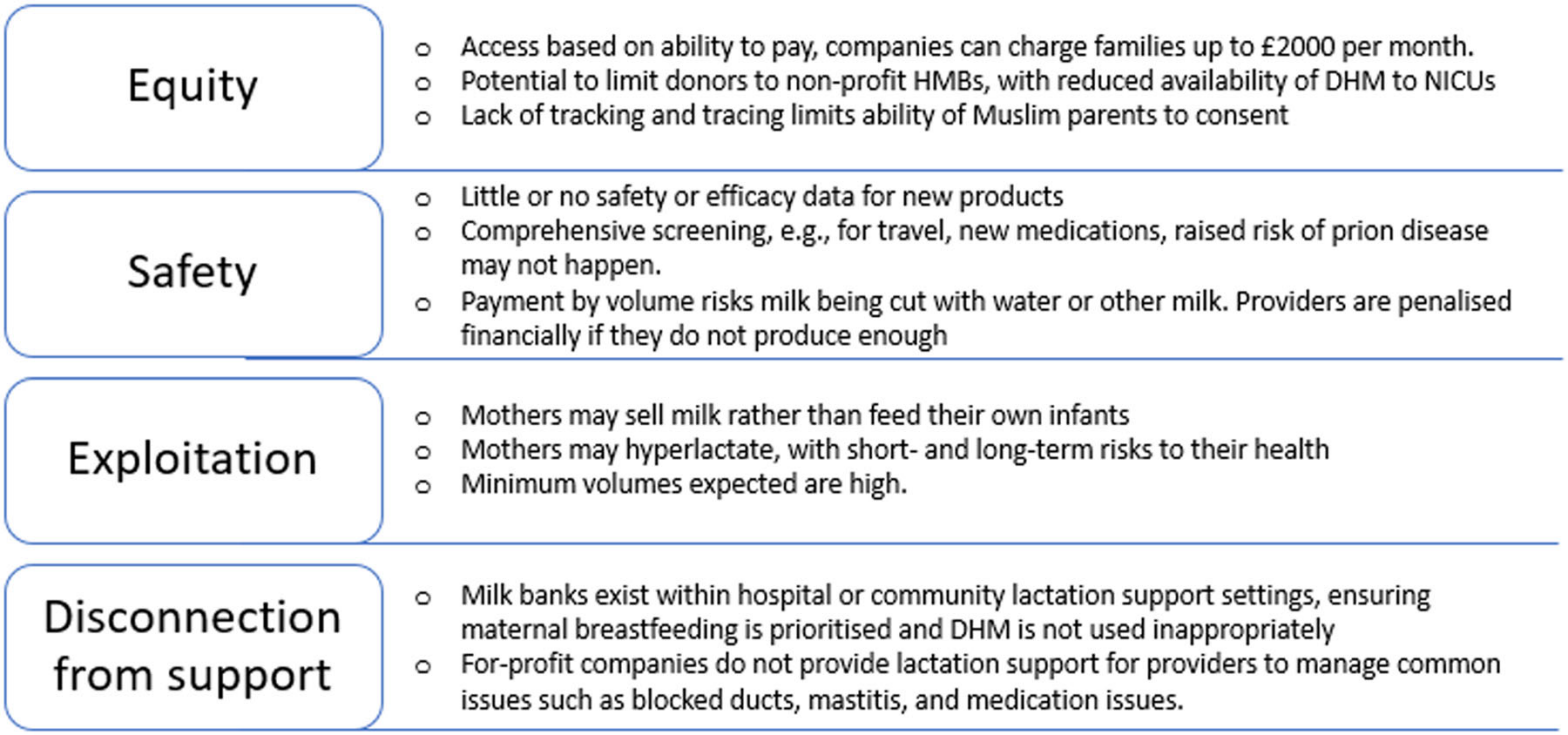
Principle risks of human milk commercialisation.

Survey responses enabled the creation of a global map of regulatory frameworks related to HM (Figure 1), with additional information from recently published data from Europe (Klotz et al., 2022). Of the 12 organisations researched, five were US based, three were located in the United Kingdom and 1 in Australia, all jurisdictions in which HM falls under the food regulators' oversight (Figure 2).

## 4 DISCUSSION

The number and scope of PMC is increasing globally. Companies are seeking to create profit from real or perceived gaps in nutritional products, adopting strategies and phraseologies from the nonprofit sector that seek to highlight the lifesaving benefits of DHM and the altruistic basis of milk donation. In part through the lack of regulation, FPC are now aiming to or already selling pasteurised HM and HM‐derived products into health care systems and directly to the general public. Such activities could be perceived as beneficial in terms of expanding the innovation and development of HM‐based products, which need investment. However, in practice, the consequences of predicted risks in terms of diminished equity, safety compromise and the potential exploitation of women have developed as a result of introducing financial remuneration to providers and from the profit‐making motivation of the companies involved. Furthermore, Principle five from the WHO guidance on the donation and management of blood, blood components and other medical products of human origin, as HM is defined, states that ‘Policies governing compensation to persons who provide biological materials for use as medical products of human origin should seek to guard against the exploitation of vulnerable individuals and promote equity in donation. The best way to achieve these goals is to adhere to a policy of financial neutrality, in which persons who donate their biological materials for use as medical products of human origin should neither benefit nor lose financially as a result of the donation. Countries should ensure that the burden of donating these materials does not fall primarily on economically disadvantaged groups (WHO, 2017). Further discussion is therefore warranted on how women and other lactating people can best be supported to become milk donors, while minimising the risk of exploitation and physical harm (Table 3).

At this point, the health benefits of HM‐based fortifiers compared to bovine milk‐based fortifiers are unclear and may be marginal (Brown et al., 2016; Eibensteiner et al., 2019; O'Connor et al., 2018). When offset against the potential harms of commercialisation in the sector, in addition to the volume of HM required to make products that would reduce broader equity in access to pasteurised DHM, the results need to be put in context.

Throughout all our consideration is that the woman who becomes a milk donor/milk provider should have access to the best level of lactation and emotional support. Milk donation is seldom a straightforward process, requiring usually at least a daily time commitment, training in terms of expressing milk and of hygiene, tracking of temperatures and expenses related to equipment and storage. Milk donors may need support for pathology related to lactation, including mastitis, blocked ducts and hyperlactation. FPC have no incentive to help support a woman to manage an over‐abundant milk supply down to desirable levels, but nonprofit milk banks see the ethical duty to the milk donor as an imperative (Hartmann, 2017). In particular need of both practical and emotional support are bereaved donors, for whom milk donation can have multiple benefits if optimally supported (Kennedy et al., 2017).

In the context of HM banking's decades‐long track record of producing safe products (Tyebally Fang et al., 2021), it is worth questioning the need for increased technological investment into HM banking where trust relationships can mitigate the need to rely on DNA matching or additional checks (Keim et al., 2015; St‐Onge et al., 2015), and whether these processes benefit safety, or add a layer of cost that health care end‐users (patients or systems) have to cover. Further research into this could provide important evidence for organisations seeking to balance safety and cost. A general lack of transparency in production processes by PMCs could raise questions about end‐product safety.

Monetary compensation can benefit mothers, particularly those who do not have the option for paid maternity leave and/or have few financial resources. Nonprofit HMBs, however, do not provide donors with financial incentives to avoid additional risks that include issues relating to equity, safety, exploitation and disconnection from lactation support (Table 3). One example not studied in this current research included Ambrosia, a US‐based company recruiting milk providers who pumped in two 3‐h shifts, 6 days a week. Journalist reports in 2017, shortly before the Cambodian government intervened to ban the export of HM, stated these mothers may not have been able to meet their own child's nutrition needs fully (Bindel, 2017; Wong, 2017). While the slippery slope argument is often overused (Fumagalli, 2020), such examples from recent past supported by survey responses from HMBs globally in this report imply that the slippery slope argument applied to HM commercialisation has already led to real‐world exploitation, and regulatory consideration is now needed on a global level.

### 4.1 Options for classification and regulation

HM does not easily fit into a single category—it can be defined and therefore regulated as a food, medical product, nutritional therapy or as a unique class (PATH, 2019a). Policymakers therefore have several options for the regulation of pasteurised HM and HM‐derived products, but with caveats in the application of each. The regulation of HM as a food ensures the processing stages meet safety requirements. However, by such regulations only relating to the processing steps, both the recruitment, screening and duty of care to the donor and the usage of DHM are omitted. The NICE Clinical Guideline developed in the United Kingdom in 2010 produced recommendations for every step of the milk donation process, including informing the health care system on equity of access (NICE, 2010), but like all guidance, the NICE Guideline has no statutory powers. Furthermore, food labelling requirements are likely not appropriate for DHM from individual donors, as opposed to pooled milk from several donors, which is common in the United States and some European countries, as a result of its highly variable nutritional composition, and difficulty in calculating recommended daily intake values from individual donations.

Given the limits on market size and cost limitations on health care services, PMCs are unlikely to build a profitable business model within a single country. Some countries have implemented legislation for import/export licensing. For example, the Indian and Cambodian governments both acted to legislate against the export of HM after the development of PMCs within their countries within the last 5 years (Wong, 2017). The Indian government's move to prevent the export of procured milk to the Australian market may have limited the original business model of NeoLacta, leading a new PMC to be established in the United Kingdom, which lacks this legislation and currently regulates HM under the Food Standards Agency.

In terms of alternative methods of regulation available to policymakers beyond food, HM could be regulated as a medicine, medical product of human origin (MPHO), which includes blood, tissues and cells, or a separate class altogether. MPHO are defined by the WHO as ‘substances derived wholly or in part from human biological materials and intended for clinical application’, and services regulated in this manner are based on the principles of respect, beneficence, equity and avoidance of harm. According to Article 21 of the Oviedo Convention (Convention for the Protection of Human Rights and Dignity of the Human Being with regard to the Application of Biology and Medicine: the Convention on Human Rights and Biomedicine [ETS No. 164]), ‘the prohibition on making the human body and its parts as such a source of financial gain’ is an absolute right enshrined in European law. Living donor MPHO legislation exists to safeguard both the donor and the provider, and ensure desired levels of safety, quality and efficacy. In line with WHO rules on substances of human origin (WHO, 2017), HM as an MPHO can neither be sold nor donated, limiting commercialisation. Countries that regulate HM as a MPHO focus on controls for transmissible diseases and bacterial content as well as on agreed underlying ethical principles, in addition to inspection protocols, donor and recipient registries and adverse event reporting. Stipulations placed by the regulation of HM as a MPHO would place additional costs on the nonprofit milk bank sector. These may ultimately impact DHM supply and ongoing service function, potentially resulting in the closure of smaller HMBs. It is noteworthy that the only nonprofit HMB surveyed to produce a shelf‐stable product (the Marmande Milk Bank) operates in France, a nation that has to date excluded the formation of any PMCs through its adopted MPHO regulatory framework. Further research would be greatly beneficial in understanding whether French governmental funding and regulatory support has fostered this product innovation in France, and what other regulators and nonprofits could learn from this process.

Secondly, milk can be regulated as a medicine, but only Slovakia has adopted this approach to date (Klotz et al., 2022). HM is difficult to regulate as a medicine mainly due to its compositional variability, with milk varying between expressions at different times, days, seasons and even throughout the course of a feed. While the use of pooling of milk and ultrafiltration processes can result in close control of macronutrient content, micronutrients would be more challenging to standardise as required, a leading issue in the production of HM fortifiers.

Thirdly, regulatory bodies have the option to create a separate, HM‐specific regulatory framework as a separate class from MPHO, but incorporating all of the relevant legislation for the relevant protections of MPHO and food regulatory frameworks.

Lastly, is it possible for HM to be regarded as a breast milk substitute? Given the potential for HM products to undermine maternal breastfeeding when used outside of a programme of optimal lactation support (Williams et al., 2016), could the for‐profit market also be considered in an amendment to the WHO on the marketing of breast milk substitutes, known as ‘The Code’. The Code implicitly recognises that health workers, women and families are susceptible to direct and indirect marketing strategies (Rollins et al., 2016; WHO, 1981).

It is notable that despite DHM sharing similar attributes with blood, organs and other substances of human origin, HM is principally regulated alongside foodstuffs in these countries (Cohen, 2017). This regulatory decision affects how organisations operate in the industry, particularly by placing emphasis on processing and preparation of product and lessen the necessity for donor welfare measures. The exception from the operations researched was the Marmande Lactarium in France, which operates as part of the public health care system, as do all HMBs in France. As such, it falls under the regulatory oversight of the Agence Nationale de Sécurité du Médicament et des Produits de Santé (ANSM) (Figure 1) (Cohen, 2017), the French regulator for all medical products and human tissues.

It is notable that the PMCs analysed are located in countries without a governing regulatory body for HM as a medical product of human origin (Figure 1). This can lead to PMCs claiming to follow local, transnational (e.g., US Food and Drug Agency), and ISO regulatory standards. However, these claims of self‐regulation are not supported by external monitoring agencies. As a comparator in the nonprofit HMB sector, the Da Nang milk bank, the first to be established in Vietnam, is an example of those without formal regulatory oversight. Created by a partnership between international charitable NGOs as well as local and national departments of health, the milk bank is operating primarily in a medical context rather than one of food safety (Mansen et al., 2021). The development of international discourse around DHM regulation, and the issues faced by the Da Nang milk bank as it grows, may determine the future regulatory standards chosen by Vietnam.

Finally, a positive response to the rise in HM commercialisation would be for further government and philanthropic investment in nonprofit HMB capacity and innovation. Nonprofit HMBs should be encouraged to keep the care of women and infants at the centre of the decision‐making process, acting as centres of lactation support where donated milk is neither bought nor sold (PATH, 2019b; Reimers & Coutsoudis, 2021). As seen with the national programme in Brazil, established in 1998, nonprofit HMBs have the potential to act as a public health vehicle that cannot only support the health outcomes of women and infants but also change perceptions around breastfeeding among the wider population (Fonseca et al., 2021).

### 4.2 Limitations

This study was limited by the online nature of the survey, which was necessary because of the limitations for other forms of contact as a result of the COVID‐19 pandemic and the pressing need for rapid information collection. The study lacked direct contact with PMCs, and further research should aim to solicit information and opinions from the for‐profit sector. Furthermore, a more comprehensive body of work should now be performed, including translated materials, which facilitate structured interviews and focus groups of key stakeholder groups, including parents, health care professionals, milk bank teams and policymakers, to understand the landscape more fully. A clearer understanding of the commercialisation and commodification of HM would help to inform the development of global minimum standards in HM banking (Shenker et al., 2020; Tyebally Fang et al., 2021).

## 5 CONCLUSIONS

There is a need for a comprehensive source of knowledge and resources about the HMB industry, such as technical procedures, funding considerations and ethical concerns. GAMBA has the opportunity to fill this gap with the formalisation of its network. As the HM industry evolves rapidly and with the additional pressures from the COVID‐19 pandemic, GAMBA recognises the importance of a global network to share knowledge and resources, and work towards developing best practice statements that encompass both technical and ethical standards, including Modern Slavery Statements, for member HMB to adopt into local practice. These may be used alongside country readiness tools developed by PATH and Alive and Thrive to guide the development of new HMBs (Mansen et al., 2021; PATH, 2019a), and guide collaborations with for‐profit organisations that adhere to the same standards. This work highlights the commonalities and differences between for‐profit and nonprofit HMB sectors by analysing business model components, resulting in key considerations for GAMBA members, the WHO and national regulatory bodies.

## AUTHOR CONTRIBUTIONS

Gillian Weaver and Natalie Shenker conceived the study. Natalie Shenker, Jonathan Linden, Betty Wang, Claudia Mackenzie, Alex Pueyo Hildebrandt, Jacqui Spears, Sushma Nangia and Danielle Davis designed the methodology and conducted the survey and data analysis. All authors contributed to the writing of the paper and approved the final draft.

## CONFLICTS OF INTEREST STATEMENT

G. W. and N. S. are cofounders of the Human Milk Foundation. G. W. is cofounder and past president of the European Milk Bank Association and cofounder and past Chair of the UK Association for Milk Banking.

## Supporting information

Supporting information.

## Data Availability

The data that support the findings of this study are available from the corresponding author upon reasonable request.
