# Supplementary material for: Comparison between the for‐profit human milk industry and nonprofit human milk banking: Time for regulation?
Source: Matern Child Nutr. 2023 Oct 13;20(1):e13570. doi: 10.1111/mcn.13570 (PMC10749996; doi:10.1111/mcn.13570)
Supplement: Supplementary file 1 — Supporting information. [file MCN-20-e13570-s001.pdf]

GAMBA commercialisation survey

Start of Block: About the study

Q2 This survey is being sent to members of the Global Alliance of Milk Banks and Associations as part of a research evaluation through Imperial College London of the scope of human milk commercialisation worldwide. We are very grateful for your support in completing this survey. Please answer as many questions as you are able to. If you are unable to provide the exact answers to some questions please give as much information as you can.   The aim of the survey is to find out about the global activities in the for-profit human milk sector and about the commercialisation and commodification of human milk. The questions are all about the provision and the availability of human milk and human milk products in the country where you live. If you are able to provide additional details about such products in other countries please include them in the 'any other information' section at the end.
All information from the survey will be used to gain an insight into the extent and spread of commercial human milk companies globally and about their operations, including how milk providers are contacted, any payments made and what products are available.   The information received from the completed surveys will be collated and the results made available to all who complete the survey. We will aim to share the information to individuals who agree to be contacted for follow up, online and in academic published papers.   Ethics approval has been waived for the collection of this data as it falls under the remit of a service evaluation, and no patient information is being asked for. The survey should only take around 15-20 minutes to complete. You will have the option for this to be provided anonymously, and if you give your contact details for your information to be removed at a future date by emailing natalie.shenker09@imperial.ac.uk.

End of Block: About the study

Start of Block: About you

Q3 Do you want to answer anonymously?

- ○ Yes (1)
- ○ No (2)

Display This Question:

If Do you want to answer anonymously? = Yes

Q50 Which country to you live in / are answering for?

____

Display This Question:

If Do you want to answer anonymously? = No

Q49 Please complete the following:

- ◯ Name (1) ____
- ◯ Professional role (2) ____
- ◯ Address (3) ____
- ◯ Address 2 (4) ____
- ◯ City (5) ____
- ◯ State (6) ____
- ◯ Country/Region (7) ____

Display This Question:

If Do you want to answer anonymously? = No

Q5 Please give your contact number and email address if you are happy to be contacted by the study team for follow-up information:

- ◯ Phone number (1) ____
- ◯ Email address (2) ____

Q7 Do you currently work in the following sectors:

- ▢ Non-profit milk bank (government/ Ministry of Health operated) (1)
- ▢ Non-profit milk bank (operated by local or regional healthcare services) (6)
- ▢ Non-profit milk bank (independent) (2)
- ▢ For-profit milk bank sector (3)
- ▢ Other (please specify) (5) ____

End of Block: About you

Start of Block: Contact with for-profit companies

Q55 Have you been contacted by representatives of for-profit human milk companies?

- ○ Yes (3)
- ○ No (4)

Display This Question:

If Have you been contacted by representatives of for-profit human milk companies? = Yes

Q10 If yes, please give details including when you were contacted and why?

____

____

____

____

____

Q11 How many for-profit human milk companies have a base/site in the country in which you live?

____

Q14 Please enter as many details as possible for the largest company operating in your country:

- ○ Company name (1) ____
- ○ Year when started operating, if known (2) ____
- ○ Business address, location (3) ____
- ○ Website (4) ____

Q52 Does this company make / sell: (tick all that apply)

- ▢ Frozen human milk (1)
- ▢ Liquid human milk (2)
- ▢ Modified human milk product (e.g., increased fat content) (3)
- ▢ Freeze-dried (lyphilised) human milk (4)
- ▢ Frozen human milk fortifier (5)
- ▢ Dried human milk fortifier (6)
- ▢ Human milk cream products (7)
- ▢ Other (please specify) (8) ____

Q57 Would you like to add information about a second company?

- ○ Yes (1)
- ○ No (2)

Skip To: Q17 If Would you like to add information about a second company? = No

Q54 Please enter as many details as you can for the next company:

- ○ Company name (1) ____
- ○ Year when started operating, if known (2) ____
- ○ Business address, location (3) ____
- ○ Website (4) ____

Q58 Does this company make / sell: (tick all that apply)

- ☐ Frozen human milk (1)
- ☐ Liquid human milk (2)
- ☐ Modified human milk product (e.g., increased fat content) (3)
- ☐ Freeze-dried (lyphilised) human milk (4)
- ☐ Frozen human milk fortifier (5)
- ☐ Dried human milk fortifier (6)
- ☐ Human milk cream products (7)
- ☐ Other (please specify) (8)

Q59 Would you like to add information about a third company?

- ○ Yes (1)
- ○ No (2)

Skip To: Q17 If Would you like to add information about a third company? = No

---

Q56 Please enter as many details as possible for this company operating in your country:

○ Company name (1) ____

○ Year when started operating, if known (2) ____

○ Business address, location (3) ____

○ Website (4) ____

Q60 Does this company make / sell: (tick all that apply)

- ▢ Frozen human milk (1)
- ▢ Liquid human milk (2)
- ▢ Modified human milk product (e.g., increased fat content) (3)
- ▢ Freeze-dried (lyphilised) human milk (4)
- ▢ Frozen human milk fortifier (5)
- ▢ Dried human milk fortifier (6)
- ▢ Human milk cream products (7)
- ▢ Other (please specify) (8)

Q64 Would you like to add information about a fourth company?

○ Yes (1)

○ No (2)

Skip To: Q17 If Would you like to add information about a fourth company? = No

Q62 Please enter as many details as possible for this company operating in your country:

- ○ Company name (1) ____
- ○ Year when started operating, if known (2) ____
- ○ Business address, location (3) ____
- ○ Website (4) ____

Q63 Does this company make / sell: (tick all that apply)

- ▢ Frozen human milk (1)
- ▢ Liquid human milk (2)
- ▢ Modified human milk product (e.g., increased fat content) (3)
- ▢ Freeze-dried (lyphilised) human milk (4)
- ▢ Frozen human milk fortifier (5)
- ▢ Dried human milk fortifier (6)
- ▢ Human milk cream products (7)
- ▢ Other (please specify) (8)

Q68 Would you like to add information about a fifth company?

- ◯ Yes (1)
- ◯ No (2)

Skip To: Q17 If Would you like to add information about a fifth company? = No

Q66 Please enter as many details as possible for this company operating in your country:

- ◯ Company name (1) ____
- ◯ Year when started operating, if known (2) ____
- ◯ Business address, location (3) ____
- ◯ Website (4) ____

Q67 Does this company make / sell: (tick all that apply)

- [ ] Frozen human milk (1)
- [ ] Liquid human milk (2)
- [ ] Modified human milk product (e.g., increased fat content) (3)
- [ ] Freeze-dried (lyphilised) human milk (4)
- [ ] Frozen human milk fortifier (5)
- [ ] Dried human milk fortifier (6)
- [ ] Human milk cream products (7)
- [ ] Other (please specify) (8)

Q69 Would you like to add information about a sixth company?

- ○ Yes (1)
- ○ No (2)

Skip To: Q17 If Would you like to add information about a sixth company? = No

---

Q70 Please enter as many details as possible for this company operating in your country:

- ○ Company name (1) ____
- ○ Year when started operating, if known (2) ____
- ○ Business address, location (3) ____
- ○ Website (4) ____

Q71 Does this company make / sell: (tick all that apply)

- ▢ Frozen human milk (1)
- ▢ Liquid human milk (2)
- ▢ Modified human milk product (e.g., increased fat content) (3)
- ▢ Freeze-dried (lyphilised) human milk (4)
- ▢ Frozen human milk fortifier (5)
- ▢ Dried human milk fortifier (6)
- ▢ Human milk cream products (7)
- ▢ Other (please specify) (8)

Q17 Do any of the for-profit human milk companies in your country sell human milk directly to the public / parents at home?

- ◯ Yes (4)
- ◯ No (5)

Q19 Do any of the for-profit human milk companies in your country sell human milk to the public / parents at home via intermediaries?

- ◯ Yes (4)
- ◯ No (5)
- ◯ Don't know (6)

Q20 Do you have any pricing details for each product e.g., cost per 100mls / per sachet /

____

Q58 If you have any printed materials about pricing (brochures, sales lists, etc.) please upload here:

Q22 Please provide information about human milk products from for-profit companies that are imported into your country:

____

Q61 Are any products exported by for-profit companies in your country to other countries? Please give details if yes of which companies, which products and to which countries.

- ○ No (23)
- ○ Yes (24) ____

Q48 Have you been offered products/services from for-profit milk companies in exchange for your products/services?

- ○ No (1)
- ○ Yes (please give more information) (2)

____

End of Block: Contact with for-profit companies

Start of Block: Who are the providers of milk?

Q24 Who provides the human milk to the for-profit companies. Please tick all that apply:

- ▢ Mother and infant in hospital (1)
- ▢ Mother at home, infant in hospital (2)
- ▢ Mother and infant at home (3)
- ▢ Lactating hospital staff who have returned to work (4)
- ▢ Bereaved mothers (5)
- ▢ Other (please specifiy) (6)

____

Q25 Are any of the above given any money in return for their milk?

- ◯ Yes (3)
- ◯ No (5)
- ◯ Don't know (4)

Display This Question:

If Are any of the above given any money in return for their milk? =

Q26 If yes what is the nature of the payment and are there any conditions (e.g., minimum volume provided in a set time)?

____

Q27 Are any of the above given anything else in return for their milk (e.g., a non returnable breast pump, reduced fees for any services)?

- ◯ Yes (4)
- ◯ No (5)
- ◯ Don't know (7)

Display This Question:

If Are any of the above given anything else in return for their milk (e.g., a non returnable breast... = Yes

Q60 If yes, please give details:

____

Q28 How are prospective human milk providers contacted by the companies?

- ☐ Contacted directly on social media (1)
- ☐ Direct marketing in hospitals (posters, etc) (2)
- ☐ Internet advertising and website contact (3)
- ☐ Referred by healthcare professional (4)
- ☐ Contacted during pregnancy by company (5)
- ☐ Others (please specify) (6) ____

End of Block: Who are the providers of milk?

Start of Block: Non-profit sector background

Q57
In order to contextualise the growth in the for-profit human milk sector, it is important to know more about the non-profit milk banks in your country.

How many non-profit human milk banks operate in your country?

____

Q56 How are non-profit human milk banks funded in your country (tick all that apply)?

- ▢ Direct funding from government / Ministry of Health services (1)
- ▢ Local / regional healthcare services (5)
- ▢ Independent / philanthropic (2)
- ▢ Other (please specify) (6) ____

Display This Question:

If How are non-profit human milk banks funded in your country (tick all that apply)? = Independent / philanthropic

Or How are non-profit human milk banks funded in your country (tick all that apply)? =

Or How are non-profit human milk banks funded in your country (tick all that apply)? = Local / regional healthcare services

Q33 For the non-governmentally funded non-profit milk banks in your country, where does their income come from? Please tick all that apply:

- ▢ Charitable donations (1)
- ▢ Milk bank's cost recovery from provision to hospitals (2)
- ▢ Milk bank's cost recovery from provision to parents (3)
- ▢ Financial support from companies that produce breastmilk substitutes (4)
- ▢ Other (please specify) (5)

Q34 Who receives donor milk from non-profit human milk banks in your country in general (please tick all that apply):

- ☐ All preterm infants receiving neonatal care (1)
- ☐ Some preterm infants receiving neonatal care (give broad criteria here) (2) ____
- ☐ Any low birthweight infants receiving neonatal care (3)
- ☐ All sick infants receiving neonatal care, regardless of prematurity (4)
- ☐ Hospitalised well infants in case of absence of full maternal milk supply (5)
- ☐ Infants cared for at home (6)
- ☐ Other (please specify) (7) ____

Q36 Are financial charges made by the milk bank for any of the above?

- ○ Yes (1)
- ○ No (2)

Display This Question:

If Are financial charges made by the milk bank for any of the above? = Yes

Q37 If yes, please explain what these are and to whom (e.g., price per 100 ml / per ounce)?

____

End of Block: Non-profit sector background

Start of Block: Regulatory oversight

Q39 Which regulatory body has oversight over human milk banking in your country?

____

Q59 Please name the legal framework that includes the regulation of human milk collection and processing in your country, e.g., codes of food regulations, tissue, functional food, medicines, etc.

____

Q40 Is it legally permitted in your country to buy and sell breastmilk?

○ Yes (1)

○ In certain circumstances (please explain) (2) ____

○ No (3)

Q41 Is it legally permitted to sell blood in your country?

○ Yes (4)

○ No (5)

Q42 To the best of your knowledge, is the sale of other body fluids or medical products of human origin legally permitted in your country?

○ No (1)

○ Under certain circumstances (please specify) (2) ____

○ Yes (3) ____

Q61 Do you know of any other examples of companies selling products that profit from breastfeeding or the provision of human milk, e.g., nutritional analysis tools for mothers, novel forms of human milk inspired nutrition.

____

Q44 Please give any additional information you think is important to be shared below:

____

Q45 What do you think are the most important next steps in your country on the topic of human milk commercialisation, if any?

____

Q46 What do you think are the most important next steps for GAMBA on this issue, if any?

____

Q47 Are you willing to answer further questions that arise from this survey?

- ○ No (1)
- ○ Yes, by email (2) ____
- ○ Yes by phone (3) ____
- ○ Yes, by videolink (4) ____

End of Block: Regulatory oversight
